# A single-center retrospective study with 1-year follow-up after CEA in patients with severe carotid stenosis with contralateral carotid artery occlusion

**DOI:** 10.3389/fneur.2022.971673

**Published:** 2022-08-24

**Authors:** Wanzhong Yuan, Ran Huo, Kaiming Ma, Yunfeng Han, Xiaoliang Yin, Jun Yang, Xihai Zhao, Tao Wang

**Affiliations:** ^1^Department of Neurosurgery, Peking University Third Hospital, Beijing, China; ^2^Department of Radiology, Peking University Third Hospital, Beijing, China; ^3^Center for Biomedical Imaging Research, Department of Biomedical Engineering, School of Medicine, Tsinghua University, Beijing, China

**Keywords:** carotid endarterectomy, severe carotid artery stenosis, contralateral carotid artery occlusion, shunt, carotid clamp time

## Abstract

**Objective:**

To analyze the risk factors associated with adverse events after carotid endarterectomy (CEA) in patients with unilateral severe carotid stenosis and contralateral occlusion.

**Methods:**

Patients were recruited for CEA between August 2014 and February 2020. CEA was performed under general anesthesia. The carotid clamp time (CCT; long CCT: >20 min) is defined as the period between clamp-on and clamp-off for the stenotic carotid artery. The perioperative factors and postoperative adverse events were recorded. All patients were followed up for 1 year after CEA.

**Results:**

Sixty subjects (65.8 ± 7.2 years; 54 males) were included. Patients with adverse events had significantly longer CCT than those without adverse events (60% vs. 40%, *P* = 0.013). Univariate logistic regression analysis showed that a history of diabetes was significantly associated with adverse events (OR, 0.190; 95% CI, 0.045–0.814; *P* = 0.025); long CCT was significantly associated with adverse events (OR, 8.500; 95% CI, 1.617–44.682; *P* = 0.011). After adjusting for confounding factors, including age, sex, BMI, diabetes, PSV, long CCT, non–use of shunt, and history of stroke or TIA, the associations between diabetes and adverse events (OR, 0.113; 95% CI, 0.013–0.959; *P* = 0.046) were statistically significant; the associations between long CCT and adverse events (OR, 1.301; 95% CI, 1.049–1.613; *P* = 0.017) were statistically significant.

**Conclusions:**

A longer carotid clamp time (>20 min) and a history of diabetes may increase the risk of adverse events in patients with unilateral severe carotid stenosis and contralateral occlusion after CEA. With good preoperative evaluation and intraoperative monitoring, the use of shunts may not be needed intraoperatively in patients with unilateral severe carotid stenosis and contralateral occlusion.

## Introduction

Stroke is one of the leading health threats worldwide (1, 2). Carotid artery stenotic disease is a common etiology for ischemic stroke. Of patients with carotid stenosis, those with unilateral severe carotid stenosis and contralateral carotid occlusion have a higher rate of neurological symptoms than patients with the non–occluded contralateral carotid artery (3). Carotid endarterectomy (CEA), still the reference standard surgical treatment for carotid stenosis, is done by removal of the plaque and recanalization in patients with moderate to severe carotid stenosis (4). However, a meta-analysis demonstrated that patients with unilateral severe carotid stenosis and contralateral carotid occlusion had an increased incidence of mortality and stroke/transient ischemic attack (TIA) within 30 days after CEA (5).

Previous CEA studies have shown that longer operative times are associated with 30-days mortality and postoperative stroke and that a total operation time >110 min during CEA was associated with a 40% increase in cardiac complications and a 25% increase in technical complications (6). The carotid clamp time (CCT) is a critical time period for CEA surgery, but there are few studies on it. The use of shunts on the surgical side in patients with unilateral severe carotid stenosis and contralateral carotid occlusion has been the general preference in some clinical settings (7). However, there is still a debate over whether intraoperative shunts should be routinely used during CEA. Some investigators found that the use of shunts reduced postoperative cerebral ischemic and reperfusion injury (8). In contrast, other researchers reported that the use of shunts in patients with unilateral carotid severe stenosis and contralateral carotid occlusion could lead to postoperative adverse events such as ischemic or hemorrhagic stroke, cardiovascular events, hemodynamic disorders, etc (9, 10). Evaluating CCT and the usefulness of shunting during CEA in such patients would be beneficial for the optimization of CEA procedures.

The present study aimed to analyze the risk factors associated with adverse events in patients with unilateral severe carotid stenosis and contralateral occlusion after CEA.

## Materials and methods

### Study population

Sixty patients with symptomatic or asymptomatic unilateral severe carotid stenosis (70–99%) and contralateral carotid occlusion determined by computed tomography angiography (CTA) referred for CEA were recruited in this study between August 2014 and February 2020. The total number of CEA cases in the same time period was 968. The exclusion criteria were as follows: (1) severe cardiac disease (e.g., New York Heart Association class III, left ventricular ejection fraction <30%, unstable angina with intolerance to general anesthesia, recent myocardial infarction, unstable angina within the last 6 months, myocardial infarction within 30 days or progressive stroke within 3 months); (2) expected survival <2 years; (3) severe chronic obstructive pulmonary disease (e.g., arterial partial pressure of oxygen <65 mmHg, carbon dioxide partial pressure >45 mmHg, forced expiratory volume in the first second <0.5 L, forced vital capacity rate in one second <60%); (4) massive preoperative cerebral infarction (area of infarction exceeded one-third of the ipsilateral middle cerebral artery territory); and (5) history of carotid intervention treatment, such as CEA or stenting. The study protocol followed the tenets of the Declaration of Helsinki and was approved by the Medical Science Research Ethics Committee. All patients signed a written informed consent form prior to participating in this study.

### Clinical data collection

Demographics (age and sex) and traditional risk factors, including body mass index (BMI), history of hypertension, type 2 diabetes, coronary atherosclerotic heart disease, hyperlipidemia, old cerebral infarction, hyperhomocysteinemia, and history of smoking, were collected from the clinical records.

### Perioperative management

Preoperative evaluation: All patients underwent ultrasound imaging to measure the peak systolic velocity (PSV) of the carotid arteries with severe stenosis 48 h before surgery for CEA. Clinically routine CTA was also performed to evaluate the status of collateral circulation, which we used to choose whether to insert a shunt during CEA. The protocol for CTA is detailed in Supplementary Table 1.

Surgical procedure and intraoperative measurements: All the enrolled patients underwent CEA under general anesthesia by the same neurosurgeon with an experience of more than 1,000 CEA cases. All patients took aspirin (daily doses of 100 mg, oral) before the surgery and stopped taking clopidogrel 1 week before CEA. The preoperative systolic blood pressure was maintained at <180 mmHg. Intraoperatively, near-infrared spectroscopy (NIRS) and transcranial Doppler (TCD) were used to monitor cerebral regional oxygen saturation (rSO2) and middle cerebral artery blood flow velocity, respectively. Intraoperatively, intravenous heparin (single dose of 5000 μ) was routinely given before carotid artery clamping. Blood pressure was raised by 10%-20% compared to preoperation during carotid artery clamping.

Shunt (T3103AS, Edwards Lifesciences, Inc., Irvine, CA, United States) was used during CEA once patients had at least one of the following conditions: (1) incomplete circle of Willis or significant stenosis (≥50%) in the middle cerebral artery on CTA (11, 12); (2) a consistent rSO2 decrease of >20% compared with baseline by intraoperative NIRS monitoring (13); (3) sustained decrease in middle cerebral artery blood flow (>50%) compared with baseline by intraoperative TCD (14).

Deep neck dissection and manipulation of vessels were performed under a microscope. The plaques and thickened intima were carefully removed until the vessel wall was smooth. The carotid clamp time (CCT) was recorded. CCT is defined as the period between clamp-on and clamp-off for the stenotic carotid artery, which is associated with shunting if it is performed. A long CCT was defined as an operation time >20 min, based on the median of the collected data.

Postoperative management: After CEA, all patients were given heparin (2,500 IU, within 24 h), aspirin (daily dose of 100 mg, oral), and intensive statin therapy (atorvastatin, daily dose of 40 mg, oral).

### Followed-up data

All patients were followed up for 1 year after CEA. The clinical outcomes were recorded at 1 month, 6 months, and 1 year after CEA in the outpatient setting. The clinical outcomes included the following adverse events: (1) new ischemic brain lesions; (2) new cerebral hemorrhage; (3) cardiovascular events (myocardial infarction, acute heart failure, arrhythmias, etc.); (4) cervical hematoma; (5) cranial nerve injury, and (6) death.

### Statistical analysis

Continuous variables with a normal distribution are presented as mean ± standard deviation. Continuous variables with a skewed distribution are described as median and interquartile range. Categorical variables are presented as count and percentage. The clinical characteristics were compared between patients with and without adverse events using the independent *t* test, Mann–Whitney *U* test, chi-square test, or Fisher's test, as appropriate. The perioperative measurements of PSV, percentage of long CCT, and percentage of shunt use were compared between patients with and without adverse events using the Mann–Whitney U test and the chi-square or Fisher's test. Univariate and multivariate logistic regression analyses were used to calculate the odds ratio (OR) and corresponding 95% confidence interval (CI) of PSV, the use of shunts and long CCT in predicting adverse events. A two-tailed *P* value <0.05 was considered statistically significant. SPSS 20.0 (SPSS, Inc., Chicago, IL, United States) software was used for statistical analysis.

## Results

### Baseline clinical characteristics

Of all 60 recruited patients, the mean age was 65.8 ± 7.3 years, 54 (90.0%) were male, and 10 (16.7%) had adverse events. Baseline demographic and clinical characteristics are summarized in Table 1. Of all 60 recruited patients, 14 (23.3%) used shunts during CEA (incomplete circle of Willis: 5 patients; rSO2 consistently decreased >20% compared with baseline by NIRS: 6 patients; and middle cerebral artery blood flow consistently decreased >50% compared with baseline by TCD: 3 patients). As shown in Table 1, patients with adverse events had significantly longer CCT than those without adverse events (60% vs. 40%, *P* = 0.013). There were significant differences in the history of diabetes (50.0% vs. 16.0%, *P* = 0.050) and history of stroke or TIA (80.0% vs. 86.0%, *P* = <0.010) between patients with and without adverse events. Patients using shunts had a significantly longer CCT (*P* < 0.001). No significant difference was found in the use of shunts between patients with and without adverse events (60.0% vs. 80.0%, *P* = 0.339). There was no significant difference in PSV between these two groups (364.6 ± 202.1 cm/s vs. 325.4 ± 107.5 cm/s, *P* = 0.376).

**Table 1:** Baseline clinical characteristics of the study population.

	**Mean** ±**SD**, ***n*** **(%)**			***P*-value**
	**All patients (*n* = 60)**	**Adverse events (+) (*n* = 10)**	**Adverse events (-) (*n* = 50)**	
Age, years	65.8 ± 7.2	67.1 ± 8.2	65.6 ± 7.0	0.547
Gender, male	54 (90.0)	9 (90.0)	45 (90.0)	1.000
BMI, kg/m^2^	24.8 ± 2.9	23.8 ± 2.9	24.9 ± 2.9	0.277
Hypertension	35 (58.3)	7 (70.0)	28 (56.0)	0.639
Hyperlipidemia	21 (35.0)	3 (30.0)	18 (36.0)	1.000
Hyperhomocysteinemia	21 (35.0)	3 (30.0)	18 (36.0)	1.000
Diabetes	13 (21.7)	5 (50.0)	8 (16.0)	0.050
Smoke	29 (48.3)	6 (60.0)	23 (46.0)	0.644
Coronary artery disease	12 (20.0)	3 (30.0)	9 (18.0)	0.665
History of stroke or TIA	51(85.0)	8(80.0)	43(86.0)	<0.001
Long CCT	24(40)	8(80.0)	16(32.0)	0.013
Non-use of shunt	46(76.7)	6(60.0)	40(80.0)	0.339
PSV, cm/s	332.0 ± 16.4	364.6 ± 202.1	325.4 ± 107.5	0.376

BMI, body mass index; TIA, transient ischemia attack; CCT, carotid clamp time; PSV, peak systolic velocity.

### Association of CCT and shunt use with adverse events

As shown in Table 2, of all 60 patients, 10 had postoperative adverse events (16.7%), of which 6 did not use a shunt tube, 8 had long CCT, 1 had a cardiovascular event, 2 had postoperative neck hematoma, and 5 had cranial nerve injuries.

**Table 2:** Description of postoperative adverse events.

	**All patients (*n* = 60)**	**Adverse events (+) (*n* = 10)**	**Adverse events (**+**)**			
			**Long CCT**	**Non-use of Shunt**	**History of stroke or TIA**	**Diabetes**
Cardiovascular events	2 (3.3%)	2 (20%)	1 (50%)	1 (50%)	2 (100%)	1 (50%)
Cervical hematoma	2 (3.3%)	2 (20%)	2 (100%)	1 (50%)	2 (100%)	1 (50%)
Cranial nerve injury	6 (10%)	6 (60%)	5 (83.3%)	2 (33.3%)	4 (66.7%)	2 (33.3%)

TIA, transient ischemia attack; CCT, carotid clamp time.

The results of the regression analysis are shown in Table 3. Univariate logistic regression analysis showed that a history of diabetes was significantly associated with adverse events (OR, 0.190; 95% CI, 0.045–0.814; *P* = 0.025), as was long CCT (OR, 8.500; 95% CI, 1.617–44.682; *P* = 0.011). After adjusting for confounding factors, including age, sex, BMI, diabetes, PSV, long CCT, non–use of shunt, and history of stroke or TIA, the association between diabetes and adverse events (OR, 0.113; 95% CI, 0.013–0.959; *P* = 0.046) was still statistically significant, as was the associations between long CCT and adverse events (OR, 1.301; 95% CI, 1.049–1.613; *P* = 0.017). There was no significant association between PSV or the use of shunts and adverse events in univariate or multivariate logistic regression analysis (all *P* > 0.05).

**Table 3:** Association of peri-operative measurements with adverse event.

	**Univariate regression**			**Multivariate regression** ^*^		
	**OR**	**95% CI**	***P*-value**		**OR**	**95% CI**
Non-use of Shunt	0.375	0.089, 1.587	0.183		0.845	0.096, 7.467
Long CCT	8.5	1.617, 44.682	0.011		1.301	1.049, 1.613
PSV, cm/s	1.002	0.997, 1.008	0.373		1.007	0.096, 7.467
Age	1.03	0.937, 1.133	0.54		1.017	0.893, 1.158
Gender	1	0.104, 9.614	1		0.2	0.011, 3.730
BMI, kg/m2	0.876	0.690, 1.111	0.275		0.875	0.632, 1.212

CCT, carotid clamp time; PSV, peak systolic velocity.

* Adjusted for age, sex, BMI, diabetes, PSV, Long CCT, non-use of shunt and History of stroke or TIA.

## Discussion

The present study investigated the risk factors associated with adverse events after CEA in patients with unilateral severe carotid stenosis and contralateral occlusion. A longer CCT and a history of diabetes were independently associated with the risk of adverse events.

In patients with unilateral severe carotid stenosis and contralateral carotid occlusion, some surgeons prefer to use shunting during CEA. Previous studies have shown that the routine placement of shunts in cases with severe cerebral ischemia undergoing CEA can reduce cerebral reperfusion injury and postoperative stroke rates (8). However, some investigators have found that the use of shunts may lead to carotid intimal injury, air embolus, microdisruption of vulnerable plaques and formation of microemboli, and intra- or postoperative stroke due to prolonged surgical time or technical errors (9, 10). This study suggests that in such patients, the operator should predict the intraoperative CCT based on a preoperative assessment of the carotid plaque status, the patency of the intracranial collateral vessel, and the cerebral perfusion on the operative side, combined with intraoperative monitoring of cerebral ischemic events, and ultimately use these information to choose whether to shunt.

The CCT considered safe in this study was ≤20 min. Longer times may increase the risk of postoperative adverse events. Previous studies have shown that a total operation time >110 min during CEA was associated with a 40% increase in cardiac complications and a 25% increase in technical complications in the Vascular Quality Initiative (15). In addition, Aziz and colleagues found that a longer total operation time was correlated with an increased incidence of 30-days mortality and length of hospital stay in patients who underwent CEA (6). In the present study, we defined CCT as the period between clamp-on and clamp-off for the stenotic carotid artery, which is associated with shunting if it is performed. Most investigators focus on the total operation time of CEA from the beginning of the surgery to the end of the skin suture, which is not specific to the operation on the carotid artery. A good grasp of CCT first requires a detailed preoperative analysis of the carotid plaque on the operative side, including the degree of stenosis, plaque length, and intraplaque properties. It is not the case that the more severe the stenosis (>90%), the longer the plaque length (>18 mm) (16). It is also not true that the more vulnerable the plaque is (thin fibrous cap, large lipid-rich necrotic core, intraplaque hemorrhage, adventitial inflammation, and neovascularization) (17), the more necessary it is to use a shunt. The complex preoperative plaque condition may prolong the operator's CCT, and if the operator expects CCT to be > 20 min, this may increase the risk of adverse events after surgery. At this time, placement of the shunt may be considered, and the operator should make a comprehensive assessment preoperatively with his or her own surgical experience. The second is the preoperative assessment of intracranial compensation. Preoperative CTA and TCD can assess the opening of the intracranial communicating artery and the intracranial perfusion on the surgical side. If the intracranial traffic branch is not open and the perfusion on the operated side is poor, the operator may consider intraoperative placement of a shunt to shorten the CCT.

For good intraoperative monitoring, as described above, we recommend intraoperative NIRS monitoring showing a sustained decrease in rSO2 >20% from baseline and intraoperative TCD showing a sustained decrease in middle cerebral artery flow (>50%) from baseline as indications for the use of shunts. We believe this is a more important principle than CCT. Non–severe stenosis, short plaque length, and short CCT do not reduce the risk of postoperative adverse events. If both indications are present during CEA, the surgeon needs to place a shunt immediately. However, placing a shunt does not mean that the operator can now ignore the CCT. We believe that even if a shunt is placed, CCT should be kept within 20 min to reduce the occurrence of postoperative adverse events.

Our study had several limitations. First, the sample size of the present study is small. Second, only the short-term prognosis was analyzed. Long-term evaluation of adverse events after CEA is warranted to be further explored.

## Conclusion

A longer carotid clamp time (>20 min) and history of diabetes may increase the risk of adverse events in patients with unilateral severe carotid stenosis and contralateral occlusion after CEA. With good preoperative evaluation and intraoperative monitoring, the use of shunts may not be needed intraoperatively in patients with unilateral severe carotid stenosis and contralateral occlusion.

## Data availability statement

The raw data supporting the conclusions of this article will be made available by the authors, without undue reservation.

## Ethics statement

The studies involving human participants were reviewed and approved by Medical Science Research Ethics Committee of Peking University Third Hospital. The patients/participants provided their written informed consent to participate in this study.

## Author contributions

All authors contributed to the study conception and design. Material preparation, data collection and analysis were performed by WY, RH, XY, KM, and YH. The first draft of the manuscript was written by WY, TW, and XZ commented on previous versions of the manuscript. All authors read and approved the final manuscript.

## Funding

This research was supported by the Beijing Natural Science Foundation (No. 7192219) and National Natural Science Foundation of China (No. 82071308).

## Conflict of interest

The authors declare that the research was conducted in the absence of any commercial or financial relationships that could be construed as a potential conflict of interest. The reviewer YG declared a shared affiliation, with no collaboration, with the author XZ to the handling editor at the time of the review.

## Publisher's note

All claims expressed in this article are solely those of the authors and do not necessarily represent those of their affiliated organizations, or those of the publisher, the editors and the reviewers. Any product that may be evaluated in this article, or claim that may be made by its manufacturer, is not guaranteed or endorsed by the publisher.
