# Supplementary material for: A single-center retrospective study with 1-year follow-up after CEA in patients with severe carotid stenosis with contralateral carotid artery occlusion
Source: Front Neurol. 2022 Aug 24;13:971673. doi: 10.3389/fneur.2022.971673 (PMC9449422; doi:10.3389/fneur.2022.971673)
Supplement: Supplementary file 1 [file Table_1.DOC]

**Supplemental Table 1 CTA imaging protocol**

|  | Parameter of CTA |
| --- | --- |
| CT scanner | 256-row wide-body detector (GE) |
| Contrast agent | Omnipaque 350 (370 mgI/ml, GE), 40 ml**,** 4.5 ml/s |
| Saline flush | 40 ml, 4.5 ml/s |
| Trigger of bolus tracking technique | 150 HU, region of interest in the ascending aorta |
| Initiated time of image acquisition | 2 s after the threshold was reached |
| Tube voltage | 100 kV |
| Slice thickness | 3 mm |
